# Distributed Authentication Model for Secure Network Connectivity in Network Separation Technology

**DOI:** 10.3390/s22020579

**Published:** 2022-01-12

**Authors:** Na-Eun Park, So-Hyun Park, Ye-Sol Oh, Jung-Hyun Moon, Il-Gu Lee

**Affiliations:** 1 Department of Future Convergence Technology Engineering, Sungshin Women’s University, Seoul 02844, Korea; 20180912@sungshin.ac.kr (N.-E.P.); 220206035@sungshin.ac.kr (S.-H.P.); 2 Department of Convergence Security Engineering, Sungshin Women’s University, Seoul 02844, Korea; 20190923@sungshin.ac.kr (Y.-S.O.); 20190905@sungshin.ac.kr (J.-H.M.)

**Keywords:** network separation, distributed network, decentralized authentication, n-factor authentication, trust level, frame structure

## Abstract

Considering the increasing scale and severity of damage from recent cybersecurity incidents, the need for fundamental solutions to external security threats has increased. Hence, network separation technology has been designed to stop the leakage of information by separating business computing networks from the Internet. However, security accidents have been continuously occurring, owing to the degradation of data transmission latency performance between the networks, decreasing the convenience and usability of the work environment. In a conventional centralized network connection concept, a problem occurs because if either usability or security is strengthened, the other is weakened. In this study, we proposed a distributed authentication mechanism for secure network connectivity (DAM4SNC) technology in a distributed network environment that requires security and latency performance simultaneously to overcome the trade-off limitations of existing technology. By communicating with separated networks based on the authentication between distributed nodes, the inefficiency of conventional centralized network connection solutions is overcome. Moreover, the security is enhanced through periodic authentication of the distributed nodes and differentiation of the certification levels. As a result of the experiment, the relative efficiency of the proposed scheme (REP) was about 420% or more in all cases.

## 1. Introduction

Considering the increase in the scale of damage and severity of recent cyber security incidents, there is a need for systems to protect critical information in response to increasingly intelligent external security threats [1]. To satisfy this need, the government has introduced the objective of network separation through basic guidelines in national information security [2].

Network separation refers to the logical and physical separation of a business network from an external Internet network, designed to prevent external attacks by blocking the Internet connection network at the source [3]. However, using mobile storage media to deliver data from the separated network between users or other networks decreases the level of security and causes security vulnerabilities in the system [4,5]. Therefore, network connection technology is required to overcome this limitation.

The current network connection concept is a logical and physical network separation and connection method. Therefore, when either usability (convenience and performance) or security is strengthened, the other is weakened [5,6]. Thus, it has a trade-off limitation that must be solved. This limitation can cause a deterioration in service quality and security, because the number of network users and frequency of network access increase [6]. To overcome the limitations of the existing technology, a network connection solution that can improve usability, while maintaining the same level of security as a separate network, is necessary.

In this study, we solve trade-off limitations, and a distributed authentication mechanism for secure network connectivity (DAM4SNC) is proposed to ensure high security and usability in a distributed network environment simultaneously. By communicating with separated networks based on the periodic authentication between the distributed nodes, the inefficiency of conventional centralized network connection solutions is improved, considering the usability and security. The security can also be enhanced by differentiating the authentication levels based on the number of successes and distance (number of hops), because the distributed nodes in the connection network attempt to achieve a periodic authentication between the trusted and peripheral nodes.

Therefore, this study has the following contributions.

1. The essential trade-off problem of network separation technology in a distributed environment is presented, and an effective and simple model to improve it is presented.

2. DAM4SNC is introducing a secure communication structure in a separate network. In the result of the experimental, DAM4SNC showed that the relative efficiency of the proposed scheme (REP) was about 420% or more in all cases in the same authentication level compared to the existing method that does not adopt frame aggregation and trust node technology.

The rest of this paper is organized as follows. Section 2 describes the existing technology, studies, and limitations for comparison with the proposed DAM4SNC technology. Section 3 describes the DAM4SNC technology. Section 4 details the experimental setup and simulation environment and discusses the experimental results. Finally, Section 5 concludes the paper and presents areas for future research directions.

## 2. Background and Related Works

### 2.1. Background

Multi-factor authentication is a method that uses a combination of two or more authentications [7,8,9]. The conventional single authentication method involves security threats through the simple leakage of personal information or malicious hacking [10,11]. However, multi-factor authentication can enhance security using multiple authentication processes [9]. With multi-factor authentication, the number of authentications increases with the number of factors [7]. Therefore, there is a trade-off because, although security is improved, user convenience decreases.

Additionally, to improve the latency performance of data processing, an aggregation technique was introduced. Such a technique can be performed at the packet or frame level. It combines multiple packets or frames in one large bundle for transmission [12,13,14]. When the aggregation technique is performed at the Internet protocol or application layer, it is classified as a packet aggregation. If it is performed at lower layers, such as physical or medium access control layers, it is classified as a frame aggregation.

**Figure 1:**
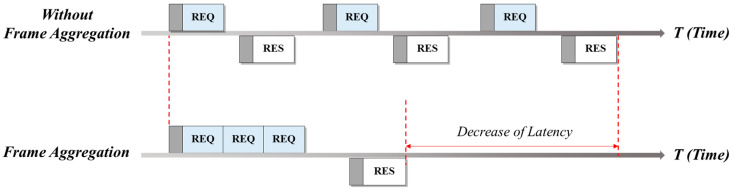
Frame aggregation.

The operation mechanism of frame aggregation is illustrated in Figure 1. Using the communication method without aggregation, an appropriate response packet (RES) is immediately sent when a request packet (REQ) is received. However, when the frame aggregation technique is applied, multiple request packets are combined and sent as one large bundle, which can be responded to by one response packet. Therefore, communication latency can be considerably improved, compared with communication without such aggregation.

Furthermore, the frame aggregation technique can integrate multiple frames into one large frame for better control of the transmission frames and to ensure efficient partial retransmissions. This technique has been proposed for next-generation wireless networks and has the advantage of reducing both transmission time and overhead [14,15,16,17].

### 2.2. Related Works

National institutions, such as the US Federal Financial Institutions Examination Council and National Institute of Standards and Technology, recommend protecting networks by separating trusted and untrusted domains [18,19]. However, a network connection is indispensable for the security patches and services of a separated network. The latency performance and security deteriorate as hardware media such as USBs are used for data exchanges between the networks [4,5].

A network connection system was designed to solve this problem, and an inter-network data transmission system was introduced to exchange data and interconnect services between the separated networking systems. However, data must also be exchanged between the separated and connected domains, wherein security must be maintained at the same level as a separate network in inter-network data transmission systems. Although inter-network data transmission techniques are being actively researched [20,21], the majority of studies do not consider both usability and security [22,23]. When the size and density of a network are larger, the security and usability are more deteriorated, owing to the structural limitations of the conventional centralized authentication and network connection technology. Therefore, safe and efficient authentication and network connection technologies are required for the era of highly dense networks based on the Internet of Things [24,25].

Additionally, most conventional IoT connection systems consist of a centralized client-server model to provide services [26,27]. However, due to the inherent characteristics of the IoTs, it risks an overhead on the central server when multiple devices are connected at the same time and communicate simultaneously. This can incur several problems, as follows: first, the system performance degrades, and a bottleneck occurs when the network traffic and number of clients increase [28,29,30,31]. Second, because the client–server model has a centralized structure, if the central server has a problem or an account of the central server is seized by an unauthorized user, it affects all the clients that belong to the same network [28,32,33].

Distributed network technologies are being studied to address this problem of centralized networks [34,35,36,37]. A distributed system was suggested that enhanced the security of the existing centralized system, solved the privacy problem of external cloud services, and improved the data integrity and security [33]. When encrypted data of the secure shell algorithm are input to a blockchain network, a verification is performed on the network to provide a fast transaction speed and data storage efficiency. However, this study has a limitation, in that it evaluates the performance only based on the blockchain network. A new distributed authentication method was proposed for distributed hospital networks using blockchain and introduced an information protection technique for the centralized system [37]. The efficiency of the model in [37] was analyzed by throughput and overhead, as well as response time. However, this study has a limitation in that the environment is limited to the blockchain and medical system. Distributed algorithms were proposed [35,36]; a centralizing solution was introduced for scalability [35], and peer-to-peer (P2P) distributed architectures and hybrid architectures were compared in terms of performance of P2P infrastructure and security [36]. However, they were also limited to the baseband functionalities (BBU) hotel location problem in the cloud radio access networks (C-RAN) environment [35], and only the effect on the network when a specific malicious attack occurred was analyzed in [36].

The previous studies had limitations, in that they can solve the problem in a limited field or use the proposed techniques only in specific environments such as the blockchain network. In this study, an authentication method for the distributed network that can be generally used in any environment is analyzed.

## 3. Proposed Scheme

The existing network connection system degrades the network separation effect because the configuration and management of the solutions are complex and difficult, owing to the structure being designed for performance and convenience rather than security. To prevent security incidents caused by the vulnerability of the network connection system, the need for a network connection system that provides both usability and security is emerging.

In this section, the DAM4SNC, ensuring high security and usability in a distributed network environment, as well as its frame format and operation method, is described.

### 3.1. DAM4SNC

**Figure 2:**
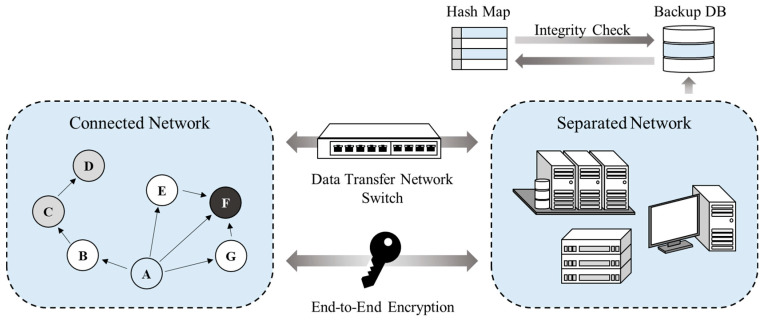
Structure of DAM4SNC.

The DAM4SNC guarantees secure network connectivity in a distributed network environment. Furthermore, it overcomes the inefficiency of the conventional network connection solutions of the centralized control method, by communicating with separated networks through authentication between the distributed nodes of the connected network. The DAM4SNC configuration designed in this study is shown in Figure 2.

Figure 2 shows the structure of a proposed system; here, each node conducts identification and authentication to improve the security in a distributed network environment, where the internal and external networks are separated. The DAM4SNC has connected and separated networks. When malicious traffic is detected through a switch between the networks, the connection to the separated network is blocked. The separated networks are logically separated from the physical networks; therefore, a higher trust level makes access more difficult and the network secure from threats. The integrity of the authentication results is maintained. Moreover, node corruption is detected using a hash map that is stored separately from the backup database, where only write is possible, contrary to a separate network where both read and write are possible.

### 3.2. Periodic Attestation and Trust Propagation

**Figure 3:**
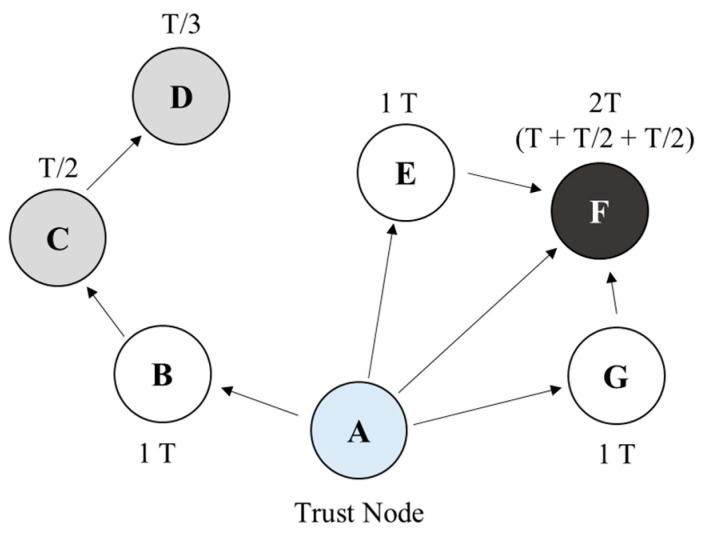
Operation of trust level.

Figure 3 shows the configuration of the connected network, comprising distributed nodes (i.e., a trust node (A) and general client nodes (B–G)). In contrast to other nodes, the trust nodes are directly managed and accessed by administrators. There is only one trust node in the local network. An administrator may be an employee with a certain level of authority, such as a data protection officer in a trusted internal network or an employee of a publicly trusted certification authority. It is assumed that the trust node in this model can be protected by the administrator by periodically accessing the node, verifying it for illegal access or contamination, and recovering immediately when a problem occurs. The client nodes increase or maintain their level of trust by periodically confirming their identity with the trust and surrounding nodes. Through this periodic confirmation, the effect of multi-factor authentication can be realized.

The distributed nodes periodically confirm their identity to the surrounding nodes, which stores the updated trust level for a unit of time.

For example, considering Figure 3, node F improves its authentication level by authenticating through trust nodes A, E, and G, which receive authentication from A. The authentication level is represented by trust level T. When a node receives direct authentication from a trust node, it receives 1 T level. When it receives authentication from a node that received authentication from a trust node, it receives an authentication level of T/(w·h), which is inversely proportional to the number of hops (h) with the trust node; here, w is the weight. The weight determines the rate of the trust level, which decreases because the distance from the trust node increases when a distributed node succeeds in the authentication through a neighbor node. In this study, modeling and simulation were applied by setting w to one to simplify the evaluation model.

For example, node D obtains an authentication level of T/3, because it receives authentication from node C at a distance of three hops from trust node A. If a node receives multiple authentications from neighboring nodes in a distributed network environment, the authentication level is determined based on the sum of the multiple authentication results. For example, according to Figure 3, client nodes E and G have a trust level of 1 T. Node F receives an authentication level of T from trust node A and an authentication level of T/2 from neighboring nodes E and G. Therefore, the level of node F becomes 2 T (=1 T + T/2 + T/2).

### 3.3. Frame Structure

**Figure 4:**
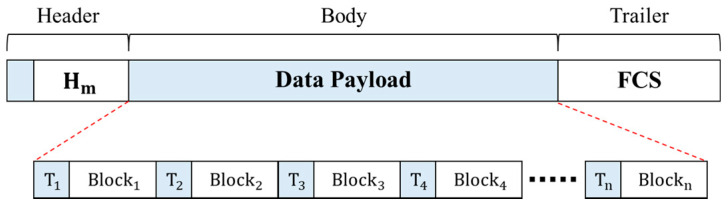
Structure of transmitted frame in DAM4SNC.

When nodes need to communicate based on the authentication level, the network connection system configures one aggregated frame with the encrypted data, hash value, and trust level of each node and sends it to the separated network. The structure of the transmitted frame is illustrated in Figure 4.

A frame comprises a header, body, and trailer. The header includes the hash map H_m_, where the hash value of each block is stored. The body area is a combination of n encrypted data of each node and the value of trust map T_n_. The trust map is an authentication result obtained when an authentication is successful, and the integrity of the authentication results is verified using the hash-based message authentication code (HMAC) algorithm. The HMAC algorithm is a special type of MAC (message authentication code) function and a representative encryption algorithm that can process input messages through a hash function, using secret keys shared with the sender and receiver [38]. By calculating each value using the shared secret key in advance and comparing it to the transmitted HMAC value, data forgery and falsification can be verified, and data integrity can be ensured [39]. Because the data of multiple nodes are grouped into a single frame for a send-off, a shorter performance time than that of the individual authentication system in a centralized network connection solution environment can be expected. Moreover, a frame check sequence was inserted into the trailer and used for error detection.

## 4. Evaluation

In this section, the proposed DAM4SNC model and a conventional centralized network connection technology model are implemented through simulation. The effectiveness of the proposed model, compared to the conventional technology, is verified based on the simulation results.

### 4.1. Evaluation Setup

The performance of the proposed DAM4SNC method and the conventional method were compared and analyzed in the same simulation environment implemented with Python 3. In the simulation model, the core function of the typical centralized method [40] was implemented for the conventional method. The simulator for these two methods was implemented in a PC environment with a 3.80 GHz Intel^®^ Core ™ i7-10700K CPU and 32 GB of RAM. The pseudocode for each model is shown in Algorithms 1–3. For simplicity in effectively comparing the proposed ideas with conventional methods, we implemented only key functions related to authentication. Latency and authentication level were measured in the same way as conventional research methodologies [40] in the simulation model. When the simulation is performed, a network is configured with randomly distributed nodes, and the conventional method is a model in which one central node authenticates and connects other nodes, and the proposed method is a model distributedly authenticated and connected by the DAM4SNC mechanism. When the core function of DAM4SNC was deactivated in the implemented simulation environment, it was confirmed that it had the same performance result as the conventional method, and then the performance was evaluated by activating the DAM4SNC functions.
**Algorithm 1. Pseudo code for DAM4SNC authentication****INPUT**: Number of nodes, Target security level each node 1 iterate (node size increases): 2  randomly set target security levels for all nodes (level 1–3) 3  **while** (until all nodes reach the target security level and transmit data): 4  // do authentication method(function) 5  authentication( ) **OUTPUT**: Latency


**Algorithm 2. Pseudo code for DAM4SNC authentication function**
**INPUT**: Number of nodes, Target security level each node 1  **def** authentication (): 2    randomly sample N_1~i_ (nodes to authenticate) 3    randomly sample M_1~j_ (nodes to be authenticated) 4    T_m_ = T_m_ + (T_n_/hop) // parallel and simultaneous authentication 5    **if** T_m_ == target_level: 6      transfer data_m_


**Algorithm 3. Pseudo code for CON authentication**
**INPUT**: Number of nodes, Target security level each node 1  iterate (node size increases): 2    set target security levels for all nodes 3    // each group of target levels has 4    // the same number of nodes of the ones of DAM4SNC’s) 5  **while** (until all nodes reach the target security level and transmit data): 6    randomly choose N (nodes to be authenticated) 7    T_n_ = T_n_ + 1 8    **if** T_n_ == target_level: 9     transfer data_n_ **OUTPUT**: Latency

Algorithms 1–3 show the pseudo codes for the simulation of the proposed DAM4SNC and conventional centralized authentication methods, respectively. The CON stands for a conventional centralized network model [40]. The conventional centralized authentication methods do not employ frame aggregation and trust node for data communications.

Regarding the DAM4SNC model, the security level required by the system can be obtained by accumulating the trust level through distributed authentication between nodes. During the experiment, the target security level was set randomly. Three security levels were defined; it was assumed that 2-, 5-, and 10-factor authentications were required to reach levels 1–3, respectively. Considering the authentication in the DAM4SNC model, a distributed node receives authentication through a neighboring trust or authenticated node, and the authenticated distributed node performs the distributed authentication for the neighboring nodes. That is, in the simulation, only inter-node data transfer is performed in the case of the existing model, and in the case of the proposed DAM4SNC, data transfer between nodes is implemented while performing trust level assignment and frame aggregation. Contrary to the conventional centralized method, which applies sequential authentication for all nodes, the proposed method authenticates each node in a repeat loop for a specified time. The trust level of the authenticated node is determined by equation T/h, depending on the number of hops for each node that is authenticated. The final trust level is determined based on the sum of the trust levels received by the authentication from the neighboring nodes for a specified time. Once the target security level is obtained, the node sends a frame to the switch, and the transmitted frames, buffered in the switch for a specified time, are aggregated and sent concurrently to the separated network. To compare the latency based on the number of nodes, some experiments are conducted while increasing the number of nodes in fixed steps, as shown in Algorithms 1–3.

Considering the conventional centralized model that is shown in Algorithm 3, the simulation is repeated while increasing the number of nodes, as in the DAM4SNC simulator, and the security level is set to the same level as that of the DAM4SNC model. Thus, the trust level is not calculated based on the number of hops; nonetheless, n authentications are conducted for n-factor authentication from the connection switch. Moreover, the data are sent when the target security level is obtained.

To compare the performance of the proposed DAM4SNC with the conventional method in the same experimental environment, the latency was measured while increasing the number of nodes in the connected network in 100 units, from 100 to 1000.

The duration of each security level required in each model can be analyzed by simulating the two models; this can be used as an evaluation index of the DAM4SNC model. When each node applies multi-factor authentication, the security of the node increases in proportion to the number of authentications. Hence, the transmission time of each node with the same number of transmissions can be determined.

The latency of each model was defined as when each node started requesting authentication to when final authentication was completed. Additionally, the difference in latency based on the security level between the two models is analyzed considering the latency of each authentication level.

### 4.2. Evaluation Results and Analysis

The conventional method of [40] and the proposed DAM4SNC were implemented in the same simulation environment. By disabling the differentiated functions such as aggregation and trust level of the DAM4SNC in this simulation environment, we confirmed the baseline of the environmental conditions for comparing DAM4SNC and conventional performance. The process of measuring and outputting the total latency and the latency of each security level for every 100 units of nodes, while increasing the nodes from 100 to 1000, was repeated 1000 times, and the averages were calculated and represented on a graph.

**Figure 5:**
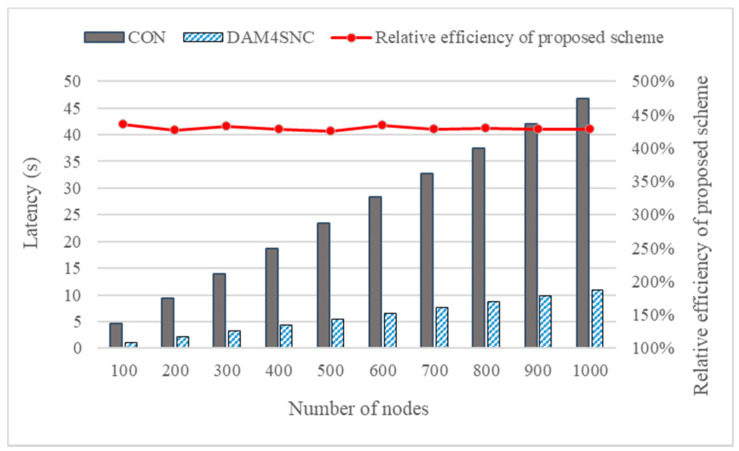
Comparison between the DAM4SNC and CON models.

The comparison results of the latency, when increasing the number of nodes in the connected network from 100 to 1000 in batches of 100 units, are shown in Figure 5. Considering Figure 5, the relative latency performance of the proposed scheme is calculated based on the formula below:

(1) $$REP\;\left(Relative\;efficiency\;of\;proposed\;scheme\right)=\frac{{\mathrm{CON}}^{\prime }s\;\mathrm{Latency}}{\mathrm{DAM}4{\mathrm{SNC}}^{\prime }s\;\mathrm{Latency}}\times 100$$

As in the experimental results, where the number of nodes were 100 and 200, the average latency of the conventional model was 4.66 s and 9.32 s. However, when DAM4SNC was applied, the latency had decreased to 1.07 s and 2.18 s, respectively. Finally, when the number of nodes was increased to 1000, the conventional model and DAM4SNC showed 46.76 s and 10.93 s, respectively, and DAM4SNC showed lower latency by about 230 s.

**Figure 6:**
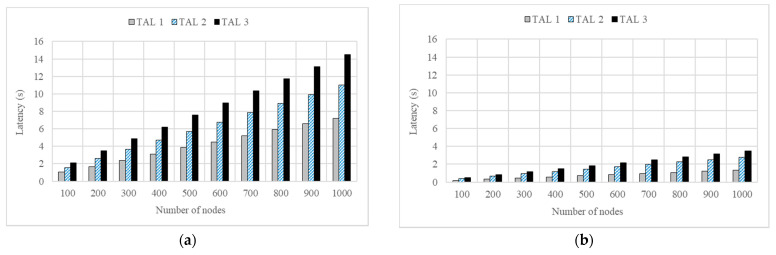
Latency based on TAL: (**a**) CON and (**b**) DAM4SNC models.

Thus, the relative efficiency of the proposed scheme (REP) calculated according to Equation (1) showed a result of about 420% or more in all cases. The difference in the latency can also be verified based on the target authentication level (TAL). Figure 6a,b show the graphs of the change in latencies based on the levels of the conventional and DAM4SNC models, respectively.

Considering Figure 6a,b, the TALs of the conventional and DAM4SNC models are expressed as TALs 1–3. It was assumed that TALs 1–3 require 2-, 5-, and 10-factor authentications, respectively. Where the number of nodes was 1000, the average latencies of the conventional model with the TAL1 to 3 were 7.20, 11.01, and 14.51 s, respectively. On the other hand, only short average latencies of 1.33, 2.79, and 3.48 s were required when the DAM4SNC model was applied.

**Figure 7:**
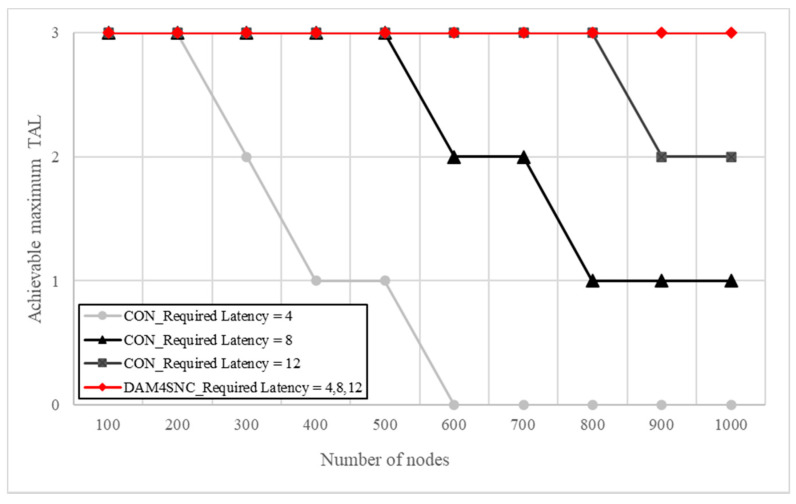
Achievable maximum TAL for the required latency conditions for each model.

Regarding the conventional model in Figure 6a, the latency increases in proportion to the security level, because the number of authentications increases when the TAL increases. This indicates that a higher multi-factor authentication is required. However, considering the proposed DAM4SNC model in Figure 6b, the latency at each security level changes at a similar level, irrespective of the security level. This means that the proposed method for a given latency requirement can guarantee a higher authentication level than the conventional method. Figure 7 is a graph of degree of achievement of TAL according to the required latency for each model.

Figure 7 shows the achievable maximum TAL for the required latency conditions over each model. The achievable maximum TAL is differentiated for the required latency in the conventional model. For example, even when the required latency was 12, the conventional model could not achieve TAL3 from when there were more than 900 nodes. In contrast, DAM4SNC could achieve TAL3 in all cases, even when the required latency was 4. Therefore, DAM4SNC was able to achieve a higher security level than the conventional model, even in a limited latency environment. When many nodes in a network send data processing requests (read/write) to the switch, each node receives the corresponding response to each request for authentication. Therefore, management frames, such as requests and responses, create an overhead in the link, reducing the total throughput. However, when the authentication level is increased through the distributed authentications among nodes in a connected network when using the proposed DAM4SNC method, the management frames are received only when the last data points are transmitted, and not during the authentication. Therefore, the overhead is lowered for the number of management frames, and an increase in the throughput can be obtained through frame aggregation. Generally, latency and throughput have a trade-off relationship. If the proposed method is used for multi-factor authentication in a distributed environment, an improvement in both latency and throughput can be expected.

## 5. Conclusions

In this study, the limitations of the conventional centralized network connection method are examined. To overcome these limitations, a DAM4SNC model is proposed. There is a need for countermeasures to external security threats owing to the continuous occurrence of cyber-attacks, and network separation technology has been designed to block such external threats. However, network separation technology has an issue with its work processing inefficiency, and the network connection model developed to solve this problem lacks security because it prioritizes efficiency.

The DAM4SNC model can improve the inefficiency of a conventional centralized network connection model because the nodes in a connected network conduct a distributed authentication in parallel, and they access the database in a separate network using the frame aggregation protocol. The distributed authentication of the DAM4SNC has the effect of multi-factor authentication based on belief propagation and enables secure authentication in a distributed network environment. Moreover, it manages the data based on the authentication level; detects malicious codes, traffic, and information forgery using the arrangement of a hash map; and includes an encrypted storage step. Consequently, the DAM4SNC model can provide a network environment with improved security, compared to the conventional centralized network connection model.

As a follow-up to this study, a mathematical analysis model for DAM4SNC will be developed, and the theoretical limits of its performance will be analyzed. Additionally, the environmental conditions for optimizing the security and performance trade-off of DAM4SNC will be analyzed through mathematical modeling and simulation.
